# Climate Drivers on Malaria Transmission in Arunachal Pradesh, India

**DOI:** 10.1371/journal.pone.0119514

**Published:** 2015-03-24

**Authors:** Suryanaryana Murty Upadhyayula, Srinivasa Rao Mutheneni, Sumana Chenna, Vaideesh Parasaram, Madhusudhan Rao Kadiri

**Affiliations:** 1 Biology Division, Council of Scientific and Industrial Research-Indian Institute of Chemical Technology, Hyderabad-500 607, India; 2 Chemical Engineering Sciences, Council of Scientific and Industrial Research-Indian Institute of Chemical Technology, Hyderabad-500 607, India; Institut Pasteur, FRANCE

## Abstract

The present study was conducted during the years 2006 to 2012 and provides information on prevalence of malaria and its regulation with effect to various climatic factors in East Siang district of Arunachal Pradesh, India. Correlation analysis, Principal Component Analysis and Hotelling’s *T^2^* statistics models are adopted to understand the effect of weather variables on malaria transmission. The epidemiological study shows that the prevalence of malaria is mostly caused by the parasite *Plasmodium vivax* followed by *Plasmodium falciparum*. It is noted that, the intensity of malaria cases declined gradually from the year 2006 to 2012. The transmission of malaria observed was more during the rainy season, as compared to summer and winter seasons. Further, the data analysis study with Principal Component Analysis and Hotelling’s *T^2^* statistic has revealed that the climatic variables such as temperature and rainfall are the most influencing factors for the high rate of malaria transmission in East Siang district of Arunachal Pradesh.

## Introduction

Malaria is a major health problem predominantly in tropical and subtropical countries [1]. According to World Health Organisation (WHO), there are about 219 million cases of malaria of which 660,000 deaths were recorded worldwide in 2010. In South East Asian region, 76% of the total malaria cases reported were mainly contributed by India (24 million cases per year), followed by Indonesia, and Myanmar [2]. North-eastern states of India are known for malarial endemicity that accounts for 10% of total malaria cases reported. *Plasmodium falciparum* in particular contributes 11% of the disease burden due to malaria. Also reports state that 46% of mortality in these states is due to malaria in the year 2007 [3–5]. Especially, the state of Arunachal Pradesh is considered to be highly endemic for malaria [6]. Transmission of malaria is known to be influenced by various climatic factors and its prevalence significantly changes due to human-pathogen relationships [7–9].

Climate change has a large impact on human health. It affects the geographic distribution of vectors and vector borne diseases and also increases the morbidity and mortality [10,11]. The influence of climatic variables on the transmission of malaria is very significant due to several factors like temperature, rainfall, wind speed and relative humidity which contribute considerably to alter the life cycle of the mosquitoes and the parasite development [12–14]. Parham & Michael reported that malaria transmission and its epidemicity are regulated mainly by the changing environmental conditions [10]. This type of studies has been carried out in most parts of the world to derive the impact of climatic variables on transmission of malaria [15–17]. However, very few studies have been conducted in India, particularly in North-eastern states such as Arunachal Pradesh [9]. High altitude areas become vulnerable to vector borne diseases like malaria as temperature increases. The state of Arunachal Pradesh experiences frequent climate variability which influences the ecology of malaria [18]. Hence, it is very difficult to predict the malarial incidence. In this context the present study is focused mainly towards investigating the relationship between meteorological factors and malaria incidence in East Siang district of Arunachal Pradesh, India. The data on climatic factors and malaria case epidemiology was collected for the years 2006–2012 and subjected for analysis. Preliminary correlation analysis was performed followed by principal component analysis (PCA). In this investigation, the contribution charts based on Hotelling’s *T* ^*2*^ statistics have been proposed for proper identification of important climatic variables that maximally influence the occurrence of malaria cases. A better understanding of the relationship between climatic factors and disease occurrence aids to improve forecasting of alterations in malaria incidence, this would shed light to concerned public health authorities to effectively distribute resources and plan the logistics for malaria control programmes.

## Materials and Methods

### Study area

**Fig 1:**
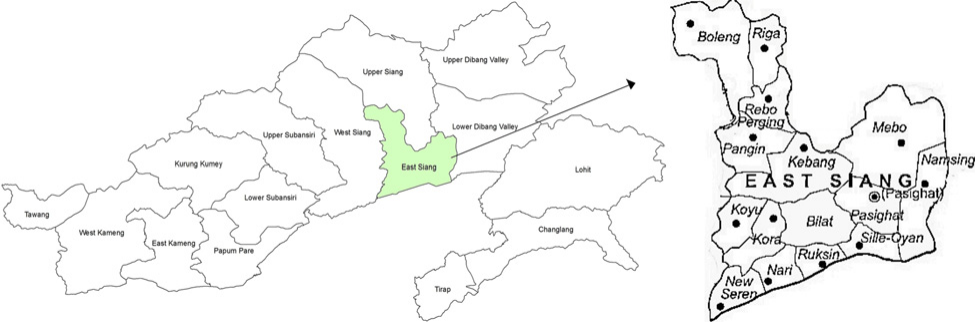
Map showing the East Siang district of Arunachal Pradesh, India.

East Siang district (Fig. 1) of Arunachal Pradesh is spread over an area of 4005 sq. km and it is situated between latitude 27 43' N & 29 20 ' N and longitude 94 42' E & 95 35' E. This district was chosen as the study area because of high incidence of malaria cases (average malaria cases was 2817 in 2006 to 279 in 2012). This district comprises of 17 Primary Health Centres (PHC)/Community Health Centres (CHC) that are equipped with diagnostic and treatment facilities for malaria and serves as reporting centre for malaria incidence under the aegis of National Vector Borne Disease Control Program (NVBDCP). The total population of East Siang district is found to be increased remarkably from 87,397 as reported by 2001 census to 99,214 as given by 2011 census over a period of 10 years. The district is bound by Upper Siang in the north, Dhemaji district of Assam in south, West Siang in the west and Dibang valley in the east. The district is mostly covered by swampy dense forest; forested terrain and perennial streams which are congenial for rapid multiplication and longevity of malaria vectors. Agriculture is the primary source of economy and most of the population in the district is engaged in agricultural activity.

### Data collection

**Epidemiological data.** Epidemiological data sets of malarial cases of East Siang District, for the years 2006 to 2012 were obtained from the Directorate of Health Services, Govt. of Arunachal Pradesh. The data was collected by using both active and passive surveillance methods which comprised of number of blood samples collected (BSC), and tested positive either for *P*. *vivax* or *P*. *falciparum* infection or mixed infection. To understand the seasonal pattern of malaria transmission, the data of malaria cases has been classified into three seasons and each season representing four months duration (March to June: summer/dry season, July to October: rainy/wet and November to February: winter/cold seasons) (Table 1).

**Table 1:** Summary of epidemiological data (number of malaria positive cases) (*PV: *Plasmodium vivax*, *PF: *Plasmodium falciparum*).

*PV	2006	2007	2008	2009	2010	2011	2012	avg/season
**summer**	1857	1485	1244	1205	1049	302	207	1049.85
**rainy**	2934	2134	1729	746	746	595	202	1298
**winter**	836	980	610	544	663	192	62	555.28
**avg/year**	**1875.6**	1533	1194.3	831.6	819.33	363	157	

*PF	2006	2007	2008	2009	2010	2011	2012	avg/season
**summer**	732	795	14	52	20	270	178	294.42
**rainy**	1654	1031	26	30	30	686	119	510.85
**winter**	438	346	25	11	1	231	70	160.28
**avg/year**	**941.33**	724	21.66	31	17	395.66	122.3	

*PV+PF	2006	2007	2008	2009	2010	2011	2012	avg/season
**summer**	2589	2280	1258	1257	1069	572	385	1344.28
**rainy**	4588	3165	1755	776	776	1281	321	1808.85
**winter**	1274	1326	635	555	664	423	132	715.57
**avg/year**	**2817**	2257	1216	862.66	836.33	758.66	279.3	

**Weather data.** Meteorological data recorded month wise on mean maximum temperature, mean minimum temperature, highest maximum temperature, lowest minimum temperature, total rainfall in the month, heavy rainfall within 24 hours, number of rainy days, mean wind speed and mean relative humidity (8.30 & 17.30 hrs) was obtained from Indian meteorological department, Government of India, Pune. These meteorological details were used to understand the effect of climatic factors on malaria.

**Weather patterns in East Siang district, Arunachal Pradesh.** The district is dominated by mountains with different altitudinal variations from 120 meters to 470 meters, above the mean sea level. The climatic conditions of East Siang district shows that the mean maximum temperature was 31°C (in the month of May) and mean minimum temperature was 12°C (January) reported during the year 2012. In this district the rainfall generally starts during the month of May and reports of high rainfall were recorded in the months from June to September. In the month of July 2012, it was observed that 1628 mm rainfall was recorded and the number of rainy days in this particular month was 23 days. During the same month it was also observed that 261 mm rainfall was recorded in 24 hours which was the heaviest rainfall reported during that period. The highest relative humidity (08.30 hrs & 17.30 hrs) was observed in the month of July (94% & 86% respectively) for the year 2012. The climatic data such as temperature, rainfall, and relative humidity of East Siang district from 2006 to 2012 is depicted in the form of graphs as supporting information (S1–S3 Figs.).

### Ethics Statement

The study received ethical approval from CSIR-Indian Institute of Chemical Technology ethical committee affiliated with the Ministry of Science and Technology, Government of India. We declare that the data on epidemiology was collected from Directorate of Health Services, Govt. of Arunachal Pradesh based on records at the PHC/CHC in Arunachal Pradesh and was analyzed anonymously; here no particular patient by name was involved.

### Data analysis

**Correlation Analysis.** Correlation analysis was performed on the data to check the statistical dependence of the climatic factors on each other as well as with the monthly incidence of malaria disease. Spearman’s correlation analysis was performed on the climatic variable data and the results of correlation coefficient (*ρ*) values are listed in Tables 2, 3 and 4.

**Table 2:** Spearman’s Correlation Matrix.

Climate variables	Mean maximum temperature	Mean minimum temperature	Highest maximum temperature	Lowest minimum temperature	Total mean rain fall	Heaviest rainfall in 24 hours	Number of rainy days	Mean wind speed	Mean relative humidity at 8.30 am	Mean relative humidity at 5.30 pm
mean maximum temperature	1	0.8940	0.9011	0.8766	0.5208	0.5470	0.4039	-0.6815	0.5128	0.4708
mean minimum temperature	0.8940	1	0.9179	0.9735	0.7738	0.7289	0.6802	-0.8365	0.7730	0.6382
highest maximum temperature	0.9011	0.9179	1	0.8918	0.7052	0.6774	0.6189	-0.7435	0.6570	0.5216
lowest minimum temperature	0.8766	0.9735	0.8918	1	0.7660	0.7317	0.6602	-0.8425	0.7591	0.6655
total mean rain fall	0.5208	0.7738	0.7052	0.7660	1	0.9155	0.9216	-0.7983	0.9021	0.6327
heaviest rainfall in 24 hours	0.5470	0.7289	0.6774	0.7317	0.9155	1	0.8301	-0.7517	0.7970	0.6187
number of rainy days	0.4039	0.6802	0.6189	0.6602	0.9216	0.8301	1	-0.7292	0.8745	0.5700
mean wind speed	-0.6815	-0.8365	-0.7435	-0.8425	-0.7983	-0.7517	-0.7292	1	-0.8421	-0.7948
mean relative humidity at 8.30 am	0.5128	0.7730	0.6570	0.7591	0.9021	0.7970	0.8745	-0.8421	1	0.7266
mean relative humidity at 5.30 pm	0.4708	0.6382	0.5216	0.6655	0.6327	0.6187	0.5700	-0.7948	0.7266	1

**Table 3:** Correlation analysis of climatic factors vrs. total number of malaria cases by year wise (*p<0.001).

Variable	Years						
	2006	2007	2008	2009	2010	2011	2012
Mean maximum temperature	0.87413*	0.78809*	0.81119*	0.44133	0.20526	0.72727*	0.49387
Mean minimum temperature	0.91608*	0.97368*	0.70403*	0.46853	0.23818	0.74126*	0.69244*
Highest maximum temperature	0.92308*	0.88617*	0.74386*	0.56392*	0.18246	0.73427*	0.59051*
Lowest minimum temperature	0.88617*	0.94571*	0.74606*	0.38246	0.23818	0.76224*	0.63047*
Total mean rain fall	0.70629*	0.72154*	0.41958	0.34266	0.3923	0.4965	0.86515*
Heaviest rainfall in 24 hours	0.74126*	0.61646	0.47552	0.29371	0.43433	0.38462	0.71103*
Number of rainy days	0.71088*	0.68134	0.19615	0.38871	0.4374	0.35275	0.83158*
Mean wind speed	-0.8601*	-0.8632*	-0.6084*	-0.3357	-0.6186*	-0.9091*	-0.7153*
Mean relative humidity at 8.30 am	0.58741	0.8193*	0.51839	0.38879	0.47368	0.56943*	0.78208*
Mean relative humidity at 5.30 pm	0.40631	0.7191	0.47369	0.19965	0.64551*	0.64906*	0.52548

**Table 4:** Correlation analysis between the climatic factors and malaria cases by season wise (*p<0.001).

Variable	Summer			Rainy			Winter		
	PF	PV	Total(PF+PV)	PF	PV	Total (PF+PV)	PF	PV	Total (PF+PV)
Mean maximum temperature	0.437*	0.254	0.454*	0.186	0.068	0.132	0.093	0.323	0.142
Mean minimum temperature	0.541*	0.2	0.547*	0.277	0.114	0.217	0.235	0.287	0.239
Highest maximum temperature	0.407*	0.3	0.415*	0.217	-0.021	0.135	0.024	0.224	0.041
Lowest minimum temperature	0.514*	0.198	0.514*	0.280	0.146	0.267	-0.032	0.183	-0.023
Total mean rain fall	0.413*	0.133	0.386*	0.099	-0.021	0.052	-0.021	0.118	-0.006
Heaviest rainfall in 24 hours	0.376*	0.176	0.365*	-0.146	-0.162	-0.195	-0.018	0.194	0.004
Number of rainy days	0.119	-0.062	0.083	-0.161	-0.177	-0.225	0.058	0.09	0.068
Mean wind speed	-0.307	0.083	-0.262	-0.281	-0.029	-0.351	0.138	0.087	0.143
Mean relative humidity at 8.30 am	0.211	-0.106	0.156	-0.07	-0.099	-0.077	-0.015	0.018	-0.015
Mean relative humidity at 5.30 pm	0.13	-0.037	0.092	0.451*	-0.091	0.354	-0.085	-0.288	-0.142

**Principal Component Analysis (PCA).** Principal component analysis (PCA) is the most widely used multivariate statistical technique for the data analysis in various applications such as pattern recognition, finance and economic trend analysis, fault detection and diagnosis etc [19, 20]. In the present study, PCA has been used for the multivariate analysis of climatic variables as well as for identification of important climatic variables that are responsible for the prevalence of malaria. The basic principle of PCA is to transform the high dimensional correlated input data linearly into a new subspace with lower dimensions in which the data is uncorrelated. It is equivalent to solving the optimization problem for finding the new orthogonal vectors which maximise the data variance in a lower dimensional subspace.

**PCA methodology.** For better illustration, the methodology of PCA is described as follows. For a typical data analysis problem being considered, the climatic variable data collected over a period of time can be stacked together into a matrix *X ∊ R* ^*Nxm*^, with *N* being the total number of samples, and *m* is the total number of measurements. *x*_*i*_ denotes *i* ^th^ sample vector as [*x*_*i*1_,*x*_*i*1_,……*x*_*im*_]^*T*^

- *Data Scaling*. Before applying PCA, the samples must be normalized such that all the variables are mean centred and vary with unit variance. The normalized variable is given by,

$$x_{ij}^{scaled}=\left(x_{ij}-{\bar{x}}_{j}\right)/\sigma_{j};i=1,2,\ldots ,N_{}andj=1,2,\ldots ,m$$ (1)

with ${\overset{-}{x}}_{j}$ be the mean and *σ*_*j*_ be the standard deviation of variable *j*.

- *Eigenvalue Decomposition*. Applying PCA is equivalent to subjecting the data covariance matrix *C* to eigenvalue decomposition or solving the following eigenvalue problem,

λP=CP (2)

The solution results in a diagonal matrix of eigenvalues, *λ* and a matrix *P* containing *m* eigenvectors. The eigenvalues λ_1_ > λ_2_………> λ_*m*_ are arranged in the decreasing order of their magnitude such that the corresponding eigenvectors are also arranged in the respective order. Each eigenvalue signifies the amount of variance expressed by the respective eigenvector. These eigenvectors can also be called as loadings, latent vectors or principal components.

- *Selection of eigenvectors*. Selection of appropriate number of principal components is one of the major issue in the development of PCA model. There are several approaches available in literature to select the optimal number of principal components (PCs) [21]. In the present work the method of cumulative percentage variance (CPV) has been used for eigenvalue selection. It is required to select the number of PCs that contribute a desired level (l*) of cumulative percentage of variance of the total variance. Then the criteria for selection of number of PCs becomes equivalent to choosing *m΄* number of components such that *m΄<< m* is given by,

$$\left(\frac{\sum_{i=1}^{m^{\prime }}\lambda_{i}}{\sum_{i=1}^{m}\lambda_{i}}\right)*100\geq l^{*}$$ (3)

The value of l* can be chosen based on the nature of data under consideration.

- *Evaluating the scores and loadings*. The matrix of selected *m΄* eigenvectors is termed as P* and can also be called as loadings matrix or principal vector matrix. Projecting the data matrix *X* on to P*gives rise to the scores matrix *T*, a matrix of new transformed variables, which are uncorrelated in nature in the reduced dimensional space.

$$T=XP^{*}$$ (4)

The scores vector is given by *t* = [*t*_1_ *t*_2_ … *t*_i_ … *t*_m΄_]^*T*^, each of its dimension *t*_*i*_ can be expressed as the linear combination of the original sample variables *x* = [*x*_1_ *x*_2_ … *x*_*m*_]^*T*^ weighted by the corresponding loading vector *p*_*i*_ as,

$$t_{i}=x_{1}p_{i}{}_{1}+x_{2}p_{i}{}_{2}+\ldots ..x_{m}p_{im}fori=1,2...m΄$$

**Feature selection through Contributions to Hotelling’s Statistics.** Hotelling’s *T* ^*2*^ index signifies the data variance in principal component sub space. Evaluating the contributions of individual variables to Hotelling’s *T* ^*2*^ index provides the means to identify important features or attributes of the data. The methodology involves mainly two steps, the first step is to evaluate the *T* ^*2*^ index and the next step is to compute the contributions of each variable to the value of *T* ^*2*^.

**Hotelling’s statistic.** Data analysis by PCA facilitates the study of deviations and processes the variable data by evaluating multivariate statistical indices. The data variation in the form of scores or transformed subspace is expressed in terms of a Hotelling’s statistic or *T* ^*2*^ statistic. This Hotelling’s *T* ^*2*^ method has been applied to various studies such as genome association studies, microarray process control and data control charts [22–24]. The Hotelling’s *T* ^*2*^ statistics can be expressed as

$$T^{2}=t^{T}\Sigma^{-1}t$$ (5)

where, *t* is the score vector representing the location of original observation *x* in the PC subspace [20].

**Contribution charts.** The basic idea of contribution charts is to quantify the role of each individual variable in representing a specific multivariate population data. Hotelling’s or *T* ^*2*^ statistic enables the representation of data variance in original measurement space as well as in the latent variable space. However, analysing *T* ^*2*^ statistic in scores space resulted by the methods such as PCA and evaluating its contribution with respect to individual variable provides the effective means to identify the important attributes of given data. Several versions of these Hotelling’s or *T* ^*2*^ statistic contribution charts are mainly used in statistical process control applications [20,25]. More recently, an improved version of the Hotelling’s or *T* ^*2*^ statistic contribution charts have been presented for fault diagnosis [26]. In the present work, the application of such Hotelling’s or *T* ^*2*^ statistic contribution charts has been extended for identifying the key climatic variables of malaria disease. The basic principle of the present contribution to Hotelling’s statistic method is to find the nearest in-control neighbour (NICN) of the observation point. The distance between these two points is used to evaluate the relative influence of each variable on the *T* ^*2*^ statistic, and further signify the contribution of individual variable in representing the data. Those variables, whose contributions are higher, can be treated as important or key features of the given data. More details about the algorithm can be referred from Viteri *et al*. [26].

## Results

### Annual trend of malaria cases (2006–2012)

**Fig 2:**
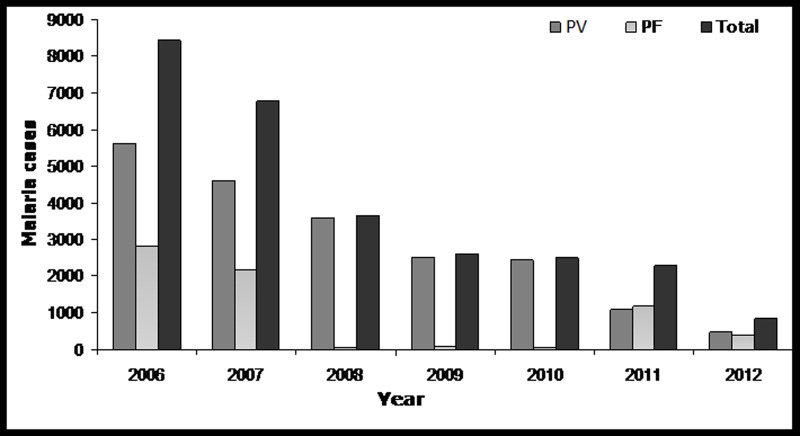
Year wise malaria cases (PF: *Plasmodium falciparum*, PV: *Plasmodium vivax*) in East Siang district of Arunachal Pradesh, India.

**Fig 3:**
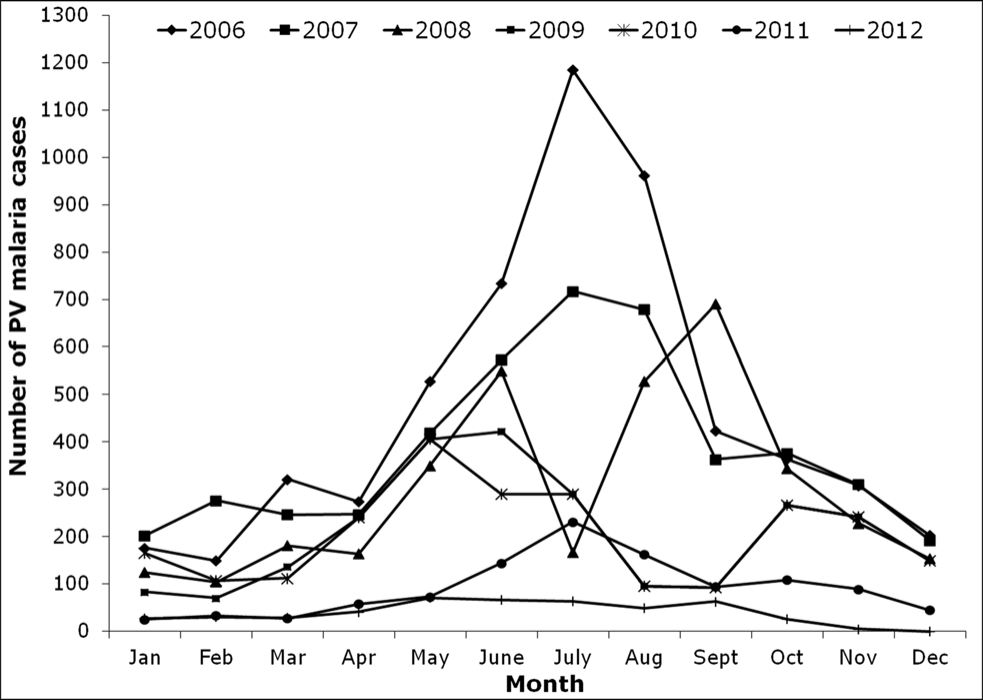
Month wise *Plasmodium vivax* (PV) malaria cases in East Siang district of Arunachal Pradesh, India.

**Fig 4:**
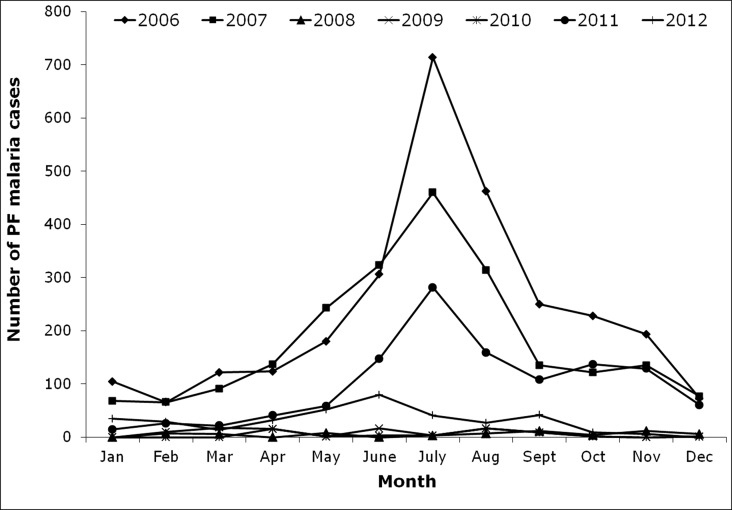
Month wise *Plasmodium falciparum* (PF) malaria cases in East Siang district of Arunachal Pradesh, India.

A total number of 27,081 malaria cases were reported during the study period (2006 to 2012). Highest number of malaria cases 8451 was recorded in the year 2006 and the prevalence rate gradually decreased with each year and reached a minimum of 838 cases in 2012, which is one tenth of the total number of cases reported in 2006 (Fig. 2). It is noted from the collected data that, the total number of malaria cases recorded per month ranged from 0 to 1899 with highest number of cases occurring in July 2006 and lowest number of cases in December 2012. The monthly epidemiological data reveals that most of the malaria cases occurred in the rainy season (Figs. 3 and 4). Based on the data, most of the malaria cases were caused by the *P*. *vivax* (20,322 cases) followed by *P*. *falciparum* parasite species (6759 cases).

### Seasonal distribution of malaria

**Fig 5:**
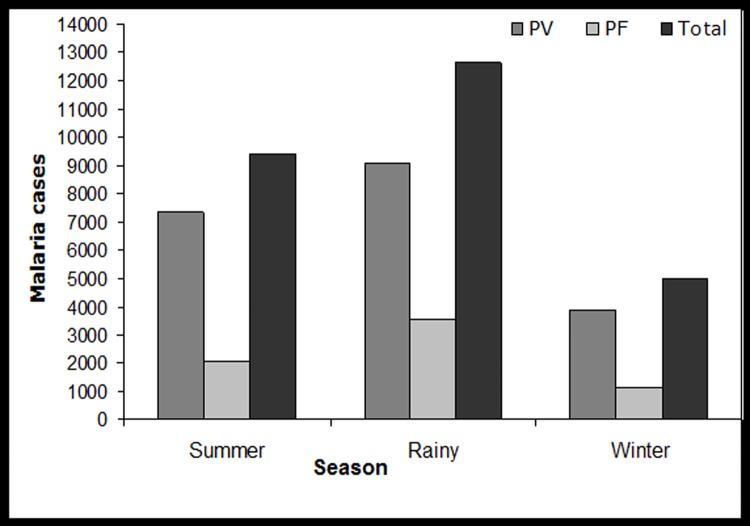
Season wise malaria cases (PF: *Plasmodium falciparum*, PV: *Plasmodium vivax*) in East Siang District of Arunachal Pradesh, India.

This study shows that malarial cases occurred in almost every month of the year however, the number of cases varied among different months. It is observed from the analysis that the average number of *P*. *vivax* cases was found to be 967.71 per year which is nearly three times higher than the number of *P*. *falciparum* cases recorded as 321.83 per year. From the seasonal distribution it is also noted that, rainy season was found to be more prone to the occurrence of malaria cases followed by summer season (Fig. 5).

### Correlation analysis of malaria with climate factors

In the present work, data analysis is performed to study the correlations existing among the climatic variables as well as the correlations between climatic variables and malaria cases. Table 2 shows that the correlation matrix of climatic variables, such as, mean maximum temperature, means minimum temperature, highest maximum temperature and lowest minimum temperature are highly correlated with each other (p< 0.001). In Table 3, the Spearman’s correlation analyses of different climatic variables with respect to the total number of monthly instances of malaria disease are listed. It is clear from the results that, all the climatic variables are positively correlated with the total number of malaria instances recorded monthly in all the years. The wind speed parameter is an exception as it is negatively correlated with the data. Similarly, the analysis is performed separately to study the relation between the climatic factors and number of malaria instances concerned to individual *P*.*falciparm*, *P*.*vivax* and total cases recorded season wise (Table 4). While analysing the season wise data it is observed that the *P*.*falciparm* cases show significant association with temperature and rainfall (Table 4).

### Multivariate analysis using PCA

**Fig 6:**
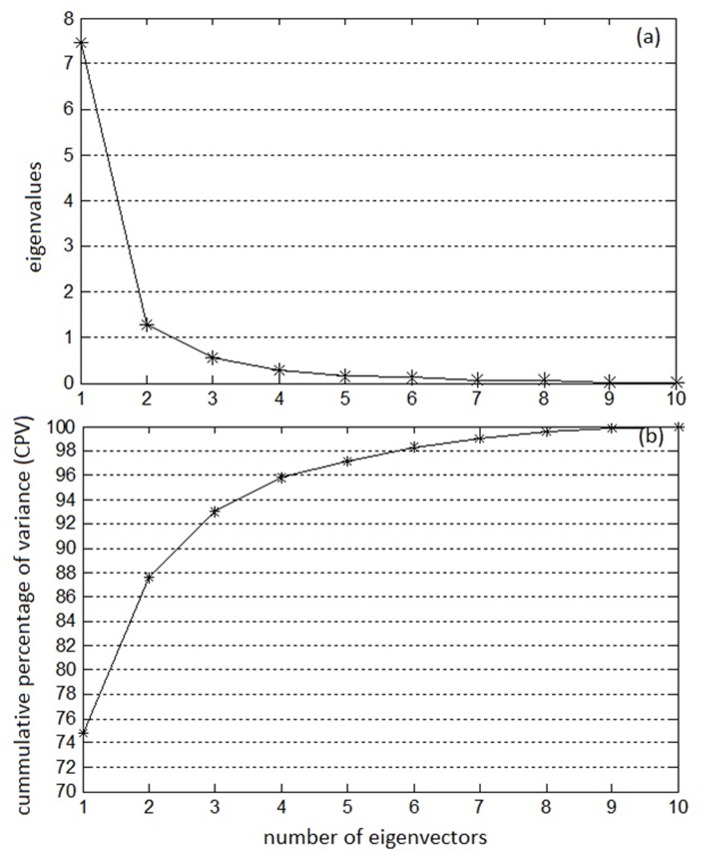
Eigenvalue analysis of climatic factor data (a) eigenvalues (b) CPV.

The monthly data of climatic factors collected during the 7 years from 2006 to 2012 is resulted in a data matrix of 84 x 10 dimensions. The data covariance matrix when subjected to PCA has resulted in a matrix of 10 eigenvectors and corresponding diagonal matrix of 10 eigenvalues which are arranged in the descending order of their magnitude. In Fig. 6a the eigenvalues are plotted across their eigenvectors signifying the extent of variance captured by individual eigenvector. It is clear from the figure that, the magnitudes of first few eigenvalues are significant and that of last few eigenvalues are nearly tend to zero, thus, revealing the importance of corresponding eigenvectors. Selection of adequate number of eigenvectors or PCs is essential for optimal data representation. Fig. 6b indicates the extent of cumulative percentage of variance (CPV) captured by the selected eigenvectors. First five eigenvectors are selected which captures a total of 97% of CPV of the total variance. Out of the five selected vectors, the first one alone is able to capture about 74.76% of total variance, and the variance captured by the remaining 2^nd^, 3^rd^, 4^th^ and 5^th^ eigenvectors are 12.79%, 5.48%, 2.76% and 1.3% respectively (Table 5). These selected eigenvectors/loadings/principal vectors are represented in Table 6. These vectors serve as the PCA model, which can be used for testing or validation.

**Table 5:** Percentage of variance captured by each eigenvector.

Eigenvector No.	Percentage of variance captured
1	74.76
2	12.79
3	5.48
4	2.76
5	1.38
6	1.11
7	0.73
8	0.55
9	0.25
10	0.18

**Table 6:** Selected Loading vector’s Matrix.

Selected eigenvectors					
Factors	*p*_1_	*p*_2_	*p*_3_	*p*_4_	*p*_5_
1	0.2865	0.5349	0.0210	-0.1218	-0.0983
2	0.3406	0.2967	0.0436	0.0884	0.0099
3	0.3206	0.3575	0.1608	0.1002	0.4034
4	0.3420	0.2695	-0.005	0.0217	-0.0935
5	0.3128	-0.3466	0.2646	-0.1324	-0.1885
6	0.3040	-0.2543	0.3417	-0.7172	0.0549
7	0.3052	-0.3411	0.3009	0.4625	0.3765
8	-0.3381	0.0713	0.2950	-0.0508	0.6321
9	0.3266	-0.2854	-0.1060	0.4037	-0.1780
10	0.2784	-0.19381	-0.7720	-0.2350	0.4550

### Selection of key climatic variables using *T* ^*2*^ contribution charts

**Fig 7:**
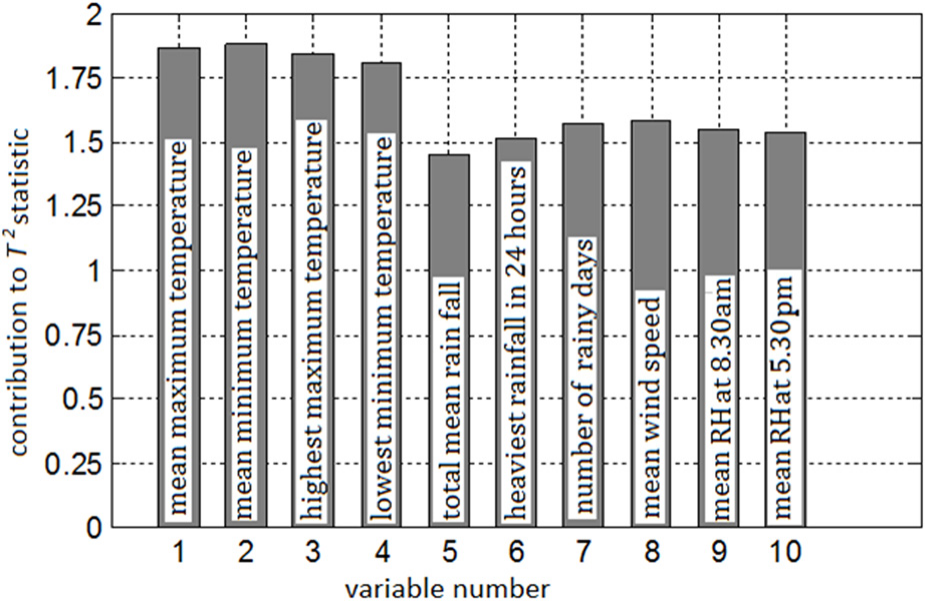
Contribution chart of climate variables.

Further, Hotelling’s *T* ^*2*^ statistic values are computed and the variable contributions to *T* ^*2*^ statistic are evaluated as per the procedure illustrated in the previous section. The result of variable contributions is represented in the form of a bar chart, and is shown in Fig. 7. The chart reveals that the most significant climatic variable which influences the malaria transmission is the temperature variable, which is found to exhibit highest contribution when compared with other climatic variables. The next important variable is the mean wind speed followed by number of rainy days. Among the four temperature variables, mean minimum temperature has shown highest contribution, followed by mean maximum, highest maximum and lowest minimum temperatures. The least significant climatic variable recorded from the study for malarial transmission happens to be total mean rainfall.

## Discussion

Malaria is a major public health problem in all North-eastern states of India and several deaths are being reported every year [4]. Earlier it was reported that, in Northeast region, *P*. *falciparum* infection accounted more than 60% and 40% was due to *P*. *vivax* [27]. In contrast to this report, we observed that higher number of malaria cases were due to *P*.*vivax* (75%) followed by *P*. *falciparum* (25%) in East Siang district of Arunachal Pradesh. The dominance of *P*. *vivax* cases over *P*. *falciparum* cases may be due to several factors like parasitic load, vector density, vectorial capacity, host parasite interaction or fresh introduction of *P*. *vivax* from nearby areas by means of migratory population to this area. It is also observed that, though the number of *P*. *vivax* cases was high throughout the study period, the transmission rates decreased gradually for the period 2006 to 2012. This decreasing trend may be due to the effective control of *P*. *vivax* cases during the study period. It is also presumed that, decrease in number of *vivax* malaria during 2008 to 2010 may be due to the exposure of Chloroquine which is an effective drug for *vivax* malaria but known for resistance to *falciparum* malaria [28]. It is interesting to note that, during this period there was a decrease in number of *P*. *falciparum* malaria cases from 2008 to 2010 but their number was found to be increased in the subsequent years, 2011 and 2012. The trend of reappearance of *P*. *falciparum* cases with high numbers clearly indicates that, there is a possibility of shift of parasitic infection in the community. Such types of trend shift of parasites was also reported with an increase in *P*. *falciparum* cases and associated with the decrease in *P*. *vivax* cases [29].

Fluctuation of malaria incidences are highly influenced with the climatic factors [30]. The climate variability impact on the incubation rate of *Plasmodium* parasites and breeding activities of *Anopheles* is considered as an important environmental contribution to malaria transmission dynamics [31,32]. There is no such report available on the transmission dynamics of malaria in East Siang district of Arunachal Pradesh, where high incidence of malaria have been observed throughout the study period. The influence of various climatic factors like temperature, relative humidity, wind speed and rainfall were analysed to understand the malaria transmission dynamics. It is observed from the study, that the disease transmission occurred throughout the year (Fig. 5), but, average to higher number of cases were recorded in rainy/wet season (1808.85) followed by summer/dry (1344.28) and winter/cold (715.42) (Table 1). The seasonal trends observed in malaria cases caused by *Plasmodium* species is illustrated in the Table 1. During the rainy season, *P*. *falciparum* cases constituted 52.9% of the patient load (n = 3576) and *P*. *vivax* cases 44.7% of the total number of cases reported in this season (n = 9086). Similarly studies from Tanzania showed that the majority of malaria cases occur during the rainy season [33]. The correlation analysis between climatic variables and malaria cases shows the significant association of *P*.*falciparum* cases with summer season (Table 4). The summer season contributed enough number of cases of *P*. *falciparum* (30.49%) and *P*. *vivax* (36.16%). This is due to the availability of vector habitation, existence of permanent water bodies, such as slow-flowing rivers, lakes and swamps which provide suitable breeding sites for malaria vectors. The mature mosquito survival is considerably reduced by low humidity [34], hence, malaria incidence during summer seasons is quite possible [35]. In contrast to our results, studies from North India have reported that the *P*. *falciparum* malaria cases were reported very high during the post-monsoon season [36].

While observing the monthly trend of malaria cases, high malaria transmission occurred in June/July to September/October. During the study period it was also observed that the first phase of malaria transmission started along with rainy season and reached its peak in July/August (i.e. South West monsoon June to September). The second phase of transmission started between October to December which also coincided with the North East monsoon. As Arunachal Pradesh receives less rainfall during the North East monsoon, the malarial cases were also scaled down. The third phase started from January to April where the disease transmission might have occurred due to the breeding of vectors in permanent breeding sites.

From the Spearman correlation analysis among monthly incidence of malaria with various meteorological variables, it is observed that, among all the climatic factors, the temperatures (maximum, minimum, highest maximum and lowest minimum) were found to be highly correlated with each other (Table 2). In addition to these temperature variables, the climatic factors such as rainfall and number of rainy days also showed strong positive correlation (*P*<0.001) with the number of malaria incidence (Table 3). The temperature and rainfall played a determined role in the transmission of malaria. Favorable temperature has an exponential effect on malaria parasite development as well as on the development of mosquito larvae [37]. Water temperature of the mosquito breeding site also influences the growth and development of mosquito larvae [38]. The factor of rainfall influences the transmission of malaria by creating the breeding sites and also increases the relative humidity, which is favorable for mosquito, parasite development and disease transmission [39]. On the other hand, abundant rainfall wash out the breeding sources which may lead to decrease in the mosquito population and reflects on decrease in number of malaria incidences. In earlier studies we have reported that temperature has high influence on the malaria transmission in Arunachal Pradesh [9]. In East Siang district, the maximum temperature (31°C) is relatively high, and creates ambient condition for mosquito breeding. It is also reported that the incidence of malaria increases in these areas during the rainy season with high temperatures [40,41]. Similar type of observation is also noted in this study where high number of cases was reported in rainy season with high temperature.

Though, Spearman correlation analysis signifies the nature and extent of correlations existing between any two variables, it may not be directly useful for multivariate analysis as well as for identifying the important factors that contribute for malaria propagation. Therefore to understand the influence of each climatic variable on malaria transmission principal component analysis followed by Hottelings *T* ^*2*^ based contribution charts were developed. The Fig. 6 gives the results of the eigenvalues and the variances explained by PCA using the related malaria variables. All the variances in the data is represented by 10 principal components. However 97% of the variance in the data is represented by the first five principal components. The individual contributions of the remaining 5 PCs are small and their total contribution is only 3% of the total variance. The eigenvectors with the highest eigenvalues represented the strongest correlation in the data set (Table 5). From Fig. 6a it is observed that the first eigenvector has the strongest correlation with malaria followed by 2^nd^ to 5^th^ PC. Thus, the first five PCs are selected for the PCs loading vector matrix. The loadings indicate the influence of variables in these five PC's and are presented in Table 6. A loading close to 1 indicates a very strong correlation. The Table 6 shows that the first rotated PCs are related to temperature. The highest loading is observed for lowest minimum temperature (0.3420), mean minimum temperature (0.3406) and highest maximum temperature (0.3206). The second related rotated PCs is related to mean maximum temperature (0.5349). The third, fourth and fifth rotated PCs are related to heaviest rainfall in 24 hours (0.3417), number of rainy days (0.4625) and mean wind speed (0.6321).

Apart from the correlation and principal component analysis, the data was further analyzed with Hotelling’s *T* ^*2*^ statistic contribution chart. The Hotelling’s *T* ^*2*^ statistic presented in this paper is a novel tool for analyzing climate data. The proposed *T* ^*2*^ statistic is corollary to its original counterpart developed for multivariate analyses [42]. The Hotelling's T2 contributions are developed with specific and significant role of climatic factors on malaria transmission [43]. The Hotelling's T2 statistic data clearly represents that the temperature variables show higher contribution in the represented data chart (Fig. 7), emphasizing that the temperature is an important determinant of malaria transmission. This result is in agreement with our earlier reported studies in Arunachal Pradesh state which states that temperature is the key factor for mosquito biology, parasite development and disease transmission [9]. Present study also clearly confirms that, the mean maximum and minimum temperatures are one of the important aspects for malaria transmission. In conclusion, temperature was identified as the key climatic variable influencing the transmission and propagation of malaria in this region. Furthermore, understanding the influence of daily temperature dynamics could provide new insights into malaria ecology in response to future climate change. Hence by considering this factor, suitable prediction model should be used to predict malaria cases for effective control.

## Supporting Information

S1 Fig Mean maximum temperature, mean minimum temperature, highest maximum temperature, lowest minimum temperature of East Siang district of Aruanchal Pradesh, India. (TIF)

S2 Fig Mean relative humidity of East Siang district of Aruanchal Pradesh, India during the year 2006 to 2012. (TIF)

S3 Fig Total rainfall in the month, heaviest rainfall in 24 hours and number of rainy days reported in East Siang district of Aruanchal Pradesh, India during the year 2006 to 2012. (TIF)
