# Supplementary figures and images for: Climate Drivers on Malaria Transmission in Arunachal Pradesh, India
Source: PLoS One. 2015 Mar 24;10(3):e0119514. doi: 10.1371/journal.pone.0119514 (PMC4372434; doi:10.1371/journal.pone.0119514)

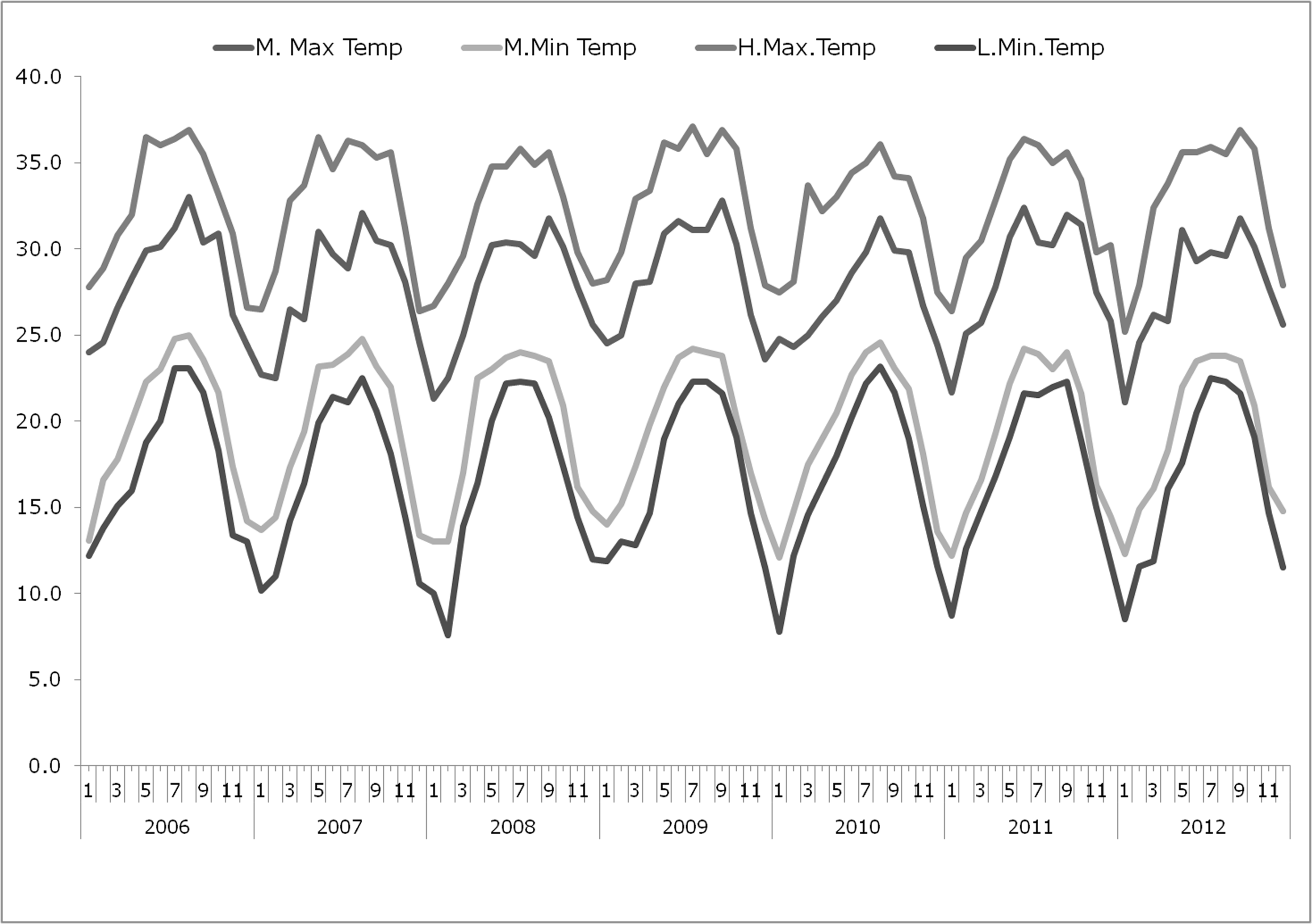

Supplement: S1 Fig — (TIF) [file pone.0119514.s001.tif]

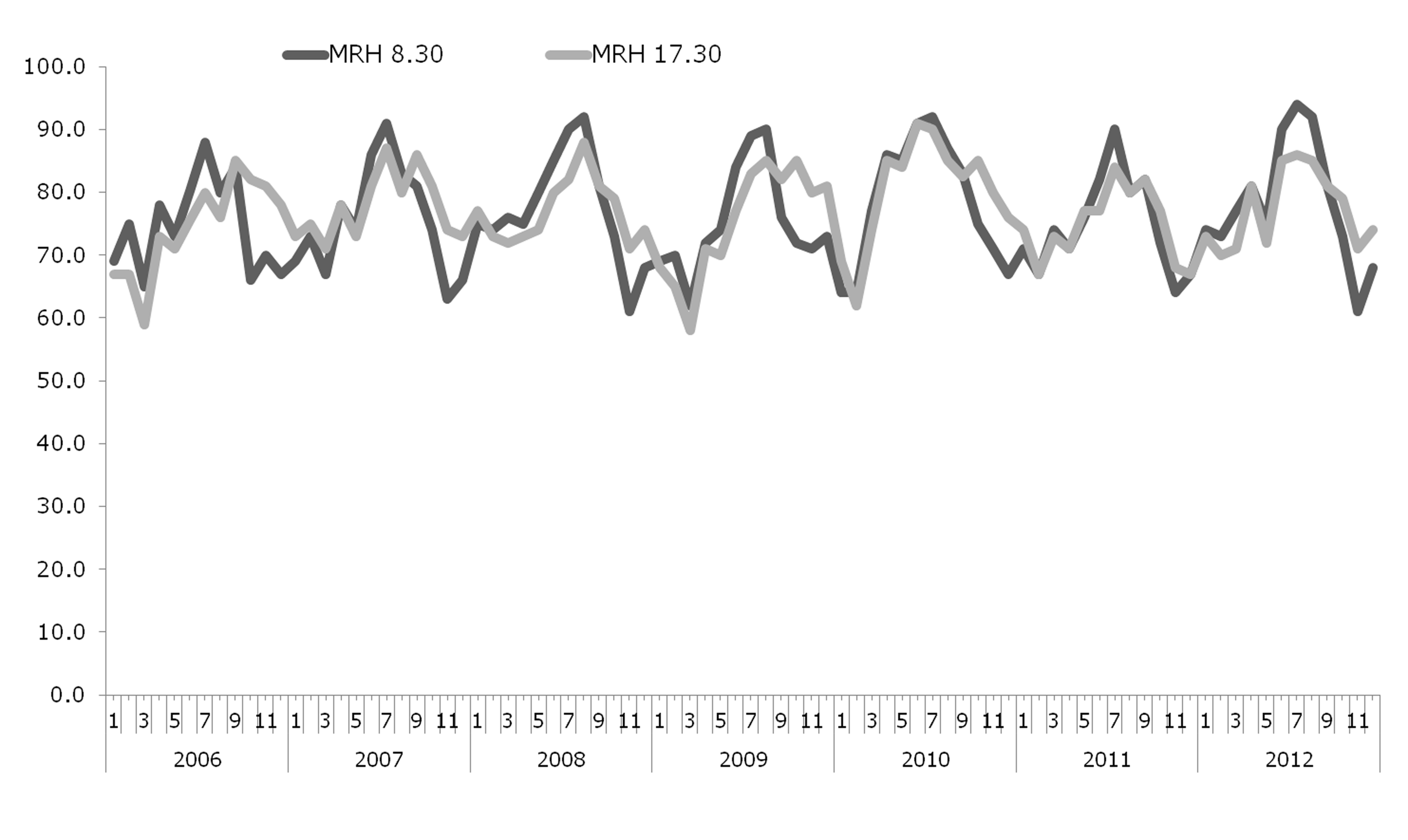

Supplement: S2 Fig — (TIF) [file pone.0119514.s002.tif]

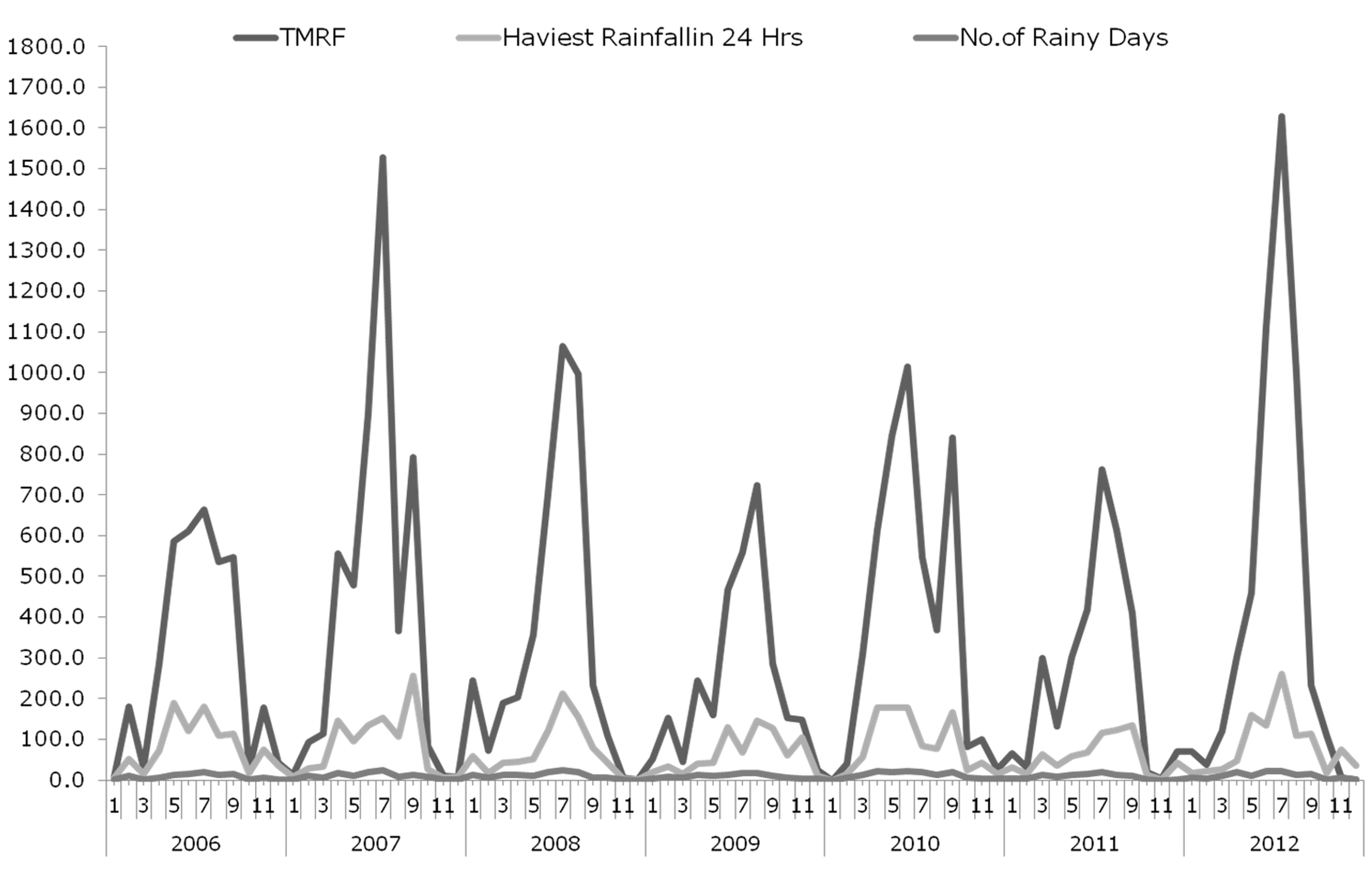

Supplement: S3 Fig — (TIF) [file pone.0119514.s003.tif]
